# Phenotypic plasticity in a population of odonates

**DOI:** 10.1038/s41598-018-26301-y

**Published:** 2018-05-31

**Authors:** Randi M. Bowman, Sharol Schmidt, Chelsea Weeks, Hunter Clark, Christopher Brown, Leigh C. Latta, Michael Edgehouse

**Affiliations:** Division of Natural Sciences and Mathematics, Lewis-Clark State College, Lewiston, Idaho USA

## Abstract

The maintenance of phenotypic plasticity within a species ensures survival through environmental flux. Plastic strategies are increasingly important given the number and magnitude of modern anthropogenic threats to the environment. We tested for phenotypic plasticity in the odonate *Argia vivida* in response to resource limitation. By limiting food availability, effectively inducing hunger, we were able to quantify shifts in agonistic behavior during intraspecific interactions. Scoring behavior in one-on-one combat trials after 1 and 4 days without food revealed phenotypic plasticity. Three classes of genotypes were identified, genotypes exhibiting either increased aggression, decreased aggression, or no phenotypic plasticity, in response to resource limitation. The variable plastic strategies in this population of odonates likely aids in maintaining fitness in fluctuating environments.

## Introduction

Phenotypic plasticity, the ability of a genotype to express multiple phenotypes in response to environmental cues, is a prevalent feature of extant biota. Plastic responses to environmental variation include changes in behavior, physiology, and morphology^1,2^. For example, some of the most well-studied models of plasticity are members of the genus *Daphnia*, which express morphological adaptations such as helmets or neck spines in response to chemicals released by their predators^3,4^. The ubiquity of phenotypic plasticity is likely because plasticity facilitates rapid, adaptive phenotypic changes in response to environmental change over very short timescales that promote population persistence (e.g.^1^). However, the maintenance of plasticity is potentially costly, and high levels of plasticity could limit evolution towards new fitness peaks^5^.

One type of phenotypic plasticity, behavioral plasticity, is a common response to changes in resource levels. In rhesus monkeys, for example, decreased food availability (leading to increased hunger) initiates lower levels of aggressive behavior^6^. More commonly, increased hunger leads to increased aggression, as seen in crayfish^7^ and the odonate *Ischnura elegans*^8^. Bergman & Moore^7^ also found that the intensity of agonistic behavior in crayfish varied depending upon the perceived value of the resource. Plastic increases or decreases in levels of aggressive behavior may be an adaptive response to environmental change in different ecological contexts. For example, decreasing aggression could be a method of conserving energy while waiting for food to become more abundant. Expending energy by increasing aggression could increase resource acquisition in a resource-depleted environment, or in environments where food is present but spatially clumped (e.g.^9,10^).

Odonates have been studied in the context of phenotypic plasticity, although literature addressing phenotypic behavioral plasticity in odonates is rather limited in scope. Most often, studies have focused on morphological change in response to predation (e.g.^11^) or in response to variation in food availability during development (e.g.^12^). Behavioral studies have concentrated on behavioral alterations in foraging behavior in response to predator presence^13–17^ and prey availability^13^. To our knowledge, no studies have addressed intraspecific behavioral plasticity in aggression along resource gradients in odonates.

Frequently, agonistic behavior in odonates is examined in the context of resource defense, including competition for mates, food items, and territory^18,19^. Unresolved is the extent to which body size plays a role in agonistic interactions. In some cases, large odonates are more aggressive^20^, which confers greater success in resource acquisition or mating^21^. In others, size is unrelated to success in resource acquisition and mating^19,22^. Regardless of the role of size, hunger appears to be a driving force behind agonistic interactions in odonates^8,18^.

To enhance our understanding of behavioral plasticity in odonates, and phenotypic plasticity in general, we investigate behavioral variation in response to food deprivation. Specifically, we tested whether depriving the odonate *Argia vivida* of food for prescribed periods of time elicits changes in intraspecific aggression consistent with behavioral plasticity. The population of odonates utilized here does not have significant predator-induced selective pressure (common predators were absent). In the absence of predation, local selective pressures likely come from social interactions related to resource acquisition. Therefore, we are confident that results from this study stem from the influence of social interactions rather than anti-predator defense mechanisms. If hunger is a driving force behind increased agonistic interactions among odonates, and we alter the hunger level of odonates by withholding food for 1 day or 4 days, then the odonates on 4 day food deprivation regime should display higher aggression scores than those with 1 day food deprivation.

## Materials and Methods

### Animals

Vivid dancer (*Argia vivida*) damselfly naiads were collected by net along the lower 0.5 km of Tammany Creek in Hells Gate State Park near Lewiston, Idaho, USA. Collections occurred between mid-February and late March (2015–16). Although Tammany Creek experiences increases and decreases in flow depending on the time of year, it flows consistently throughout the year. Mid-February to late March were chosen as time of collection because of abundance of naiads during that time. However, considering the consistency of Tammany Creek, odonates are present year around and active both as naiads and adults. Naiads were housed individually in 50 mL clear plastic vials filled with water collected from Tammany Creek. A single 3 mm diameter wooden dowel was placed in each chamber to provide a perching surface. Chambers were capped with a natural cork stopper and naiads were numbered individually. We measured the head width (HW) and body length (BL) of each naiad with a digital microcaliper. Head width was measured as the distance across the widest part of the head. Body length was measured as the distance from the tip of the head to the end of the abdomen, excluding caudal lamellae. Naiads were maintained on a photoperiod of 14:10 (L:D) at an ambient air temperature of 22 °C. Naiads were fed one shrimp (order Mysidae) every other day. Water was changed after each feeding.

### Behavior Trials

#### Setup

*A. vivida* that fed consistently for 3 feedings were placed into two groups of 5 based on size difference in 2015 (small = HW < 3 mm, BL < 8 mm; medium = HW 3–4 mm, BL 9–13 mm; large = HW > 4 mm, BL 11–19 mm) and with no regard to size in 2016. Each group was subject to different lengths of starvation. Food was withheld from Group 1 for one day before odonates were placed in the arena, and 4 days for Group 2. Both groups were then fed on the previously described feeding regime for 1 week before food restrictions were reversed (Group 1 restricted for 4 days and Group 2 restricted for 1 day).

#### Trial procedure

The arena was a 250 ml container painted black to avoid observer interference. A 3 mm dowel was placed in the center and filled with water from Tammany Creek. Each naiad in a given group was placed in the arena with every other naiad in that group for a total of 8 trials per group. The focal naiad was placed in the arena simultaneously with a competitor from the same group for 7 minutes. We recorded the behavior of both naiads, and scored them according to Table 1. Scores in Table 1 were assigned to agonistic behaviors based on previous observations. Points were awarded each time a behavior occurred, but was counted as 2 behaviors if it occurred for longer than 4 seconds continuously. At the end of 7 minutes, naiads were removed from the trial arena, and the total score for each naiad was recorded. The water in the arena was changed following each behavior trial, and the order of naiads in the trials was haphazard such that no naiad was run twice in a row to avoid possible factors of fatigue or memory. We ran trials and collected data over the course of multiple years to eliminate any possible generational impacts. We tested for the effect of year (i.e., generation) on genotype expression, but after discovering no effect, data from all years was pooled. Behavioral data from 16 focal genotypes was analyzed using generalized linear modeling assuming a Poisson distribution as implemented by the lme4 package^23^ in Program R^24^. Analysis of covariance models with genotype, resource level, their interaction, and body length (treated as a covariate) were included. In the context of these models, behavioral plasticity is suggested by a significant interaction term. To identify specific genotypes that displayed significant behavioral plasticity we used post-hoc pairwise comparisons of the 1 day vs. 4 day body length adjusted means for each genotype. To account for multiple comparisons we adjusted p-values using the Tukey method.

**Table 1:** Aggressive behavior scores.

Behavior	Description	Score (points)
No interaction	No encounter without any evidence of awareness of other individual	0
Face	One naiad orients itself to face the other	1
Circle	One naiad circles the other	2
Caudal swing	Side-to-side movement of the caudal lamellae (Corbet^27^)	2
Forward lamellae slash	Caudal lamellae moved forward rapidly (Corbet^27^)	3
Forward lamellae slash + hold	Forward lamellae slash followed by a hold of at least 3 seconds	4
Charge	One naiad quickly advances toward the other	5
Strike	One naiad quickly extends labium towards the other	6
Strike + injury	Strike resulting in injury to the other naiad	7

## Results

**Figure 1:**
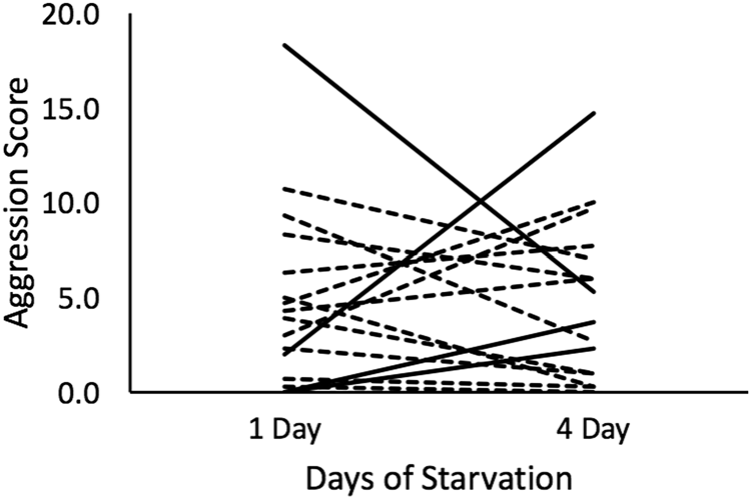
Reaction norm plot for aggression in response to changes in the duration of starvation. Individual lines represent each genotype examined in the study. Solid lines indicate genotypes that displayed a significant change in aggression across starvation treatments. Dashed lines indicate genotypes that did not display a significant change in aggression across starvation treatments.

There was significant variation in agonistic behavior among *A. vivida* genotypes (significant main effect of genotype; Table 2). The significant interaction between genotype and resource level suggests at least some of these genotypes may display significant behavioral plasticity (Fig. 1; Table 2). Post hoc pairwise comparisons indicated three different behavioral patterns across resource levels: increased agonistic behavior in response to starvation (three genotypes), decreased agonistic behavior in response to starvation (one genotype), or no change in behavior (Fig. 1; Table 3). The genotypes that displayed significant changes in agonistic behavior across resource levels indicate significant behavioral plasticity.

**Table 2:** Analysis of deviance results from a generalized linear model assuming a Poisson distribution to assess the importance of Resource Level (1 day or 4 day starvation), Genotype (16 unique genotypes), and their interaction.

Factor	df	Deviance	Residual df	Residual Deviance	p
NULL			93	707.41	
Resource Level	1	0.00	92	707.41	0.99
Genotype	15	251.79	77	455.62	<0.01
Body Length	1	5.24	76	450.38	0.02
Genotype * Resource Level	15	137.85	61	312.53	<0.01

Body Length was included as a covariate in the models.

**Table 3:** Mean aggression scores (SEM) for each genotype after one day of starvation (1 Day) and four days of starvation (4 Day).

Genotype	1 Day	4 Day
**A**	**18.3 (3.5)**	**5.3 (4.3)**
**B**	**0.0 (0.0)**	**3.7 (2.3)**
**C**	**0.0 (0.0)**	**2.3 (2.3)**
D	5.0 (4.5)	0.3 (0.3)
E	3.9 (3.0)	1.0 (1.0)
F	0.3 (0.3)	0.0 (0.0)
G	0.7 (0.3)	0.3 (0.3)
H	4.7 (2.9)	10.0 (6.4)
I	3.0 (2.5)	9.7 (2.2)
J	2.3 (1.3)	1.0 (0.0)
K	10.7 (4.8)	7.0 (0.0)
L	6.3 (5.3)	7.7 (4.1)
M	9.3 (5.8)	2.7 (0.9)
N	8.3 (6.4)	6.0 (3.2)
O	4.3 (2.0)	6.0 (2.9)
**P**	**2.0** (**1.0)**	**14.7** (**3.3)**

Genotype labels and means in bold indicate a significant difference between the 1 Day and 4 Day means based on post-hoc pairwise comparisons with p-values adjusted using the Tukey method.

## Discussion

Phenotypic plasticity allows organisms to express altered morphological, physiological, or behavioral traits in response to environmental change. Behavioral plasticity is a common response to changes in resource levels, and although it has been well-studied in odonates, studies specifically regarding changes in intraspecific agonistic behavior are lacking. Previous studies have repeatedly demonstrated hunger as a stimulus to drive changes in agonistic behavior in a wide range of taxa, including odonates^8,18^. We investigated intraspecific aggression in odonates in response to food deprivation. Although other studies (e.g.^8,25^) have investigated behavioral plasticity in odonates, ours appears to be the first to specifically address plasticity in intraspecific aggression in response to food limitation.

### Behavioral Plasticity

Our findings suggest that food deprivation causes behavioral shifts in *A. vivida* naiads. This bears similarity to Heads^13^, who described increased foraging behavior in response to increased hunger in odonates. However, the odonates in our study exhibited three different patterns of behavior in response to 4 days of food deprivation: increased, decreased, or no change in aggressive behavior (Fig. 1; Table 3). Because there are three patterns of behavior across resource levels, there must be multiple classes of genotypes of *A. vivida*, two of which express behavioral plasticity in response to food deprivation. These observations indicate that this population of odonates maintains genetic diversity for agonistic behavior, and plasticity in this behavior. Fluctuating selection acting on this population that arises due to predictable changes in the environment may maintain this plasticity in these genotypes, although this hypothesis has yet to be tested.

Similar to Heads^13,14^, our study revealed significant shifts in behavior after food deprivation. In Heads^14^, 12 days of food deprivation caused the damselfly *Ischnura elegans* to abandon dense prey-poor cover despite the presence of predators. In Heads^13^, hunger did not consistently alter penultimate instar behavior, but final instar foraging behavior was modified in that movements increased after 4 days of starvation and decreased again after 8 days. In our study, some *A. vivida* individuals experienced an increase in aggression after 4 days of starvation, while others experienced a decrease in aggression or no change at all. This variation could be an indication of the lack of additional selective pressures beyond fluctuating selection in this population. For example, there is no known predator influence on this population (R. M. Bowman & M. Edgehouse, 2016, personal observation), incurring no substantial cost to increased intraspecific aggression that could otherwise make individuals more prone to predation. Alternatively, decreased aggression could be advantageous because it conserves energy, and reducing conspecific injury could increase individual fitness.

### Observed Behavior

In our trials, agonistic behavior was only initiated with head-to-head orientation, or awareness of movement, similarly described in Edgehouse & Brown^26^. Upon this initiation, the aggressor would square its head and body to its opponent, followed with either caudal swings or strikes. This progression has been described in previous behavioral studies of larval odonates^27^. During all sets of trials, a score of 7 (strike + injury) was recorded only 4 times. It is unclear whether this agonistic behavior was defensive or predatory; however, predatory luring has been described for *A. vivida* in Edgehouse & Brown^26^. During their study it was noted that *A. vivida* use specific movements to lure the much larger *Aeshna palmata* to within striking distance and then strike in what was determined to be an intent to harm or consume. We noticed similar behaviors occurring agonistically between two *A. vivida* naiads, so it could be the same predatory action; however, with so few strike + injury scores, we hypothesize that the behavior is more likely a display than a predatory action.

The use of non-injurious signals to convey meaning and intention in agonistic interactions has been documented in many organisms (red-stag^28^; slender crayfish^29^, side-blotched lizard^30^ among others). ‘Battling’ organisms avoid harm and come away with a possible gain in fitness^31^. Our data demonstrates a similar behavioral pattern in this population of odonates. In only one of the 4 strike + injury incidents was the actual injury of a naiad observed. In this event, a single lamella was torn off of one naiad, and the aggressor stopped pursuit to consume the lamella. Because of the rare occurrence of the strike + injury behavior, we believe *A. vivida* naiads may be conveying intention while avoiding injury in agonistic interactions, possibly resulting in an overall increase in fitness. This idea is supported in other studies (e.g.^25^) that suggest that larvae avoid aggressive interactions and the associated risk of injury or death by decreasing activity and increasing monitoring of other larvae.

### Limitations

A few limitations were encountered in this study, though none would have likely affected the outcomes in a significant manner. Small sample size and a single level of food deprivation was initially concerning, because odonates were collected from one population and some died during the study. However, while our results would benefit from larger sample sizes and multiple levels of deprivation to better assess the relative frequencies of the three genotypic classes we observed, it would not have altered the primary finding that there is behavioral plasticity in this population. Having a larger sample size in future studies would make the data, and therefore our conclusions, more robust. Additionally, filming each interaction would have allowed us to identify more specific behavioral data, potentially enabling the identification of subtle cues that may not be easily visible by human observation. Finally, because we conducted this study in a lab setting rather than in the field, our results may not be reflective of what occurs in a natural environment where untold numbers of variables come into play. Supplementing this research with studies in the field would be ideal, but difficult because of common logistical problems that come with observing small organisms in an aquatic environment.

## Conclusion

In light of these observations we suggest that genotypic variation for behavior and plasticity in behavior are maintained in this population to improve resilience in the face of a fluctuating environment. When a resource (food) was limited, three behavioral patterns became apparent: increased behavior, decreased behavior, and no change. We can envision scenarios in which genetic drift maintains this phenotypic plasticity, but further investigation is needed to confirm the specific evolutionary forces acting on this population of odonates.
